# Pharmacologically induced endolysosomal cholesterol imbalance through clinically licensed drugs itraconazole and fluoxetine impairs Ebola virus infection *in vitro*

**DOI:** 10.1080/22221751.2021.2020598

**Published:** 2022-01-07

**Authors:** Susann Kummer, Angelika Lander, Jonas Goretzko, Norman Kirchoff, Ursula Rescher, Sebastian Schloer

**Affiliations:** a Center for Biological Threats and Special Pathogens, Robert Koch Institute, Berlin, Germany; b Research Group Regulatory Mechanisms of Inflammation, Institute of Medical Biochemistry, Centre for Molecular Biology of Inflammation, University of Muenster, Muenster, Germany; c Interdisciplinary Centre for Clinical Research, University of Muenster, Muenster, Germany; d Cluster of Excellence “Cells in Motion”, University of Muenster, Muenster, Germany

**Keywords:** Ebola virus, Niemann-Pick C1, itraconazole, FIASMA, fluoxetine, viral entry, endolysosomal interference

## Abstract

Ebola virus disease (EVD) is a severe and frequently lethal disease caused by Ebola virus (EBOV). The latest occasional EVD outbreak (2013–2016) in Western African, which was accompanied by a high fatality rate, showed the great potential of epidemic and pandemic spread. Antiviral therapies against EBOV are very limited, strain-dependent (only antibody therapies are available) and mostly restricted to symptomatic treatment, illustrating the urgent need for novel antiviral strategies. Thus, we evaluated the effect of the clinically widely used antifungal itraconazole and the antidepressant fluoxetine for a repurposing against EBOV infection. While itraconazole, similar to U18666A, directly binds to and inhibits the endosomal membrane protein Niemann-Pick C1 (NPC1), fluoxetine, which belongs to the structurally unrelated group of weakly basic, amphiphile so-called “functional inhibitors of acid sphingomyelinase” (FIASMA) indirectly acts on the lysosome-residing acid sphingomyelinase via enzyme detachment leading to subsequent lysosomal degradation. Both, the drug-induced endolysosomal cholesterol accumulation and the altered endolysosomal pH, might interfere with the fusion of viral and endolysosomal membrane, preventing infection with EBOV. We further provide evidence that cholesterol imbalance is a conserved cross-species mechanism to hamper EBOV infection. Thus, exploring the endolysosomal host–pathogen interface as a suitable antiviral treatment may offer a general strategy to combat EBOV infection.

## Introduction

Because of their potential of pandemic spreads, emerging infectious diseases pose serious threats to the human population. Due to globalization, climate change, and closer contact to wildlife, endemic and pandemic outbreaks have increased, resulting in a huge burden on health care systems and economics around the world. One of the deadliest infectious diseases is Ebola virus disease (EVD), with a strain-dependent average case-fatality rate ranging from 25% to 90% [1]. In 2014, the Zaire strain caused an epidemic outbreak that spread from Guinea, Liberia, Sierra Leone, and Nigeria to the rest of the world, with cases also reported in in South Africa, Western Europe, Middle East Asia, China, Canada, and the United States. The case fatality rate was 62%, and more people died during this 2014 epidemic than in all previous (documented since 1976) outbreaks combined [2]. Ebolaviruses (EBOV) belong to the family of *Filoviridae* (order: *Mononegavirales*) which is hallmarked by the filamentous shape of the lipid-enveloped viral particles that contain the non-segmented RNA-genome in negative-sense orientation [3]. Of the six known EBOV strains (Reston, Bombali, Bundibugyo, Sudan, Tai Forrest, and Zaire), the last four are the main cause of lethal hemorrhagic fever in humans and other primates [3]. The time between virus exposure and the onset of symptoms varies between two days and three weeks [4]. As the course of disease progresses, immunosuppression and a systemic inflammatory response become apparent, ultimately leading to multiorgan failure and septic shock [1].

Antiviral treatment options against EVD are very limited and primarily supportive, including (i) intravenously applied fluids and electrolytes, (ii) maintenance of oxygen status by providing oxygen therapy, and (iii) application of medications to stabilize blood pressure, and to reduce vomiting, diarrhea, fever and pain. In the past, most attempts to find a sufficient treatment failed to be licensed in humans [5,6]. Currently, the main therapeutic strategy is focusing on antibody treatment and vaccination. However, both approaches do not target all circulating Ebola virus strains. Thus, a strain-independent superior antiviral strategy is urgently needed.

Drugs targeting the host–pathogen interface rather than viral proteins are considered novel and promising antiviral approaches [7]. A compelling strategy for such an antiviral intervention is to hinder the transfer of the viral genome into the host cell. For enveloped viruses, this occurs through fusion of the viral lipid hull with cellular membranes, which enables access of the viral genome to the host cell cytosol. After the initial cellular uptake, Ebola virus reaches the endosomes, where the fusion of endosomal and viral membrane occurs [8]. A prerequisite for the fusion is the cleaving of the viral surface glycoprotein (GP) via endosomal proteases, and the direct interaction of the primed GP with the endosomal membrane protein Niemann-Pick C1 (NPC1) [9,10], which functions in the endolysosomal cholesterol egress [11]. Blocking the GP-NPC1 interaction has been suggested as a potential drug target to prevent EBOV infections [12,13]. Indeed, the widely used NPC1 small molecule inhibitor U18666A has been shown to protect cells from Ebola infection *in vitro* [14]. However, U18666A is not well tolerated [15]. We previously reported that the endolysosomal cholesterol homeostasis might be a suitable antiviral target for two highly transmittable enveloped viruses, influenza A virus (IAV) and SARS-CoV-2 [16–18] which can be targeted through repurposing of clinically licensed drugs [16,17]. Therefore, we investigated the impact of this strategy on EBOV infection in the African green monkey kidney cell line Vero E6, a widely used cell culture model for basic Ebola research [19], in MoKi cells, a recently established bat cell line culture model representing the natural host [20], and in the human lung cell line A549 [21]. Here, we present conceptual evidence that disruption of the endolysosomal cholesterol balance via repurposing of clinically well-established drugs might open new therapeutic avenues to counteract Ebola infection. Our data reveal that the antifungal triazole itraconazole, that has been described to also inhibit NPC1 [22], caused heightened endolysosomal cholesterol levels, with a concomitant decrease in EBOV infection rates. Of note, this was not only observed in the Vero E6 cells, but could also be recapitulated in MoKi cells and in the human cell line A549. Moreover, treatment with the widely used antidepressant fluoxetine [23], which belongs to the unrelated group of functional inhibitors of the acid sphingomyelinase, also disrupted the endolysosomal cholesterol balance in all three infection models and impaired Ebola infection in vitro. Therefore, targeting the Ebola-endosome interface via repurposing of well-established drugs holds promise for novel antiviral approaches to combat EBOV.

## Material and methods

### Cells and drug treatment

MoKi cells (established from *Mops condylurus* kidney cells, [20]), the African green monkey kidney cell line Vero E6, and the A549 human lung adenocarcinoma cell line were maintained in Dulbecco’s modified Eagle’s medium (DMEM, Sigma) supplemented with 2 mM L-glutamine (Thermo Fisher Scientific), 200 U/mL penicillin/100 µg/mL streptomycin (Merck), and 10% standardized fetal calf serum (FBS Superior; Merck), in a humidified incubator at 37°C and 5% CO_2_. Itraconazole (2 mg/mL, Sigma), U18666A (10 mg/mL, Biomol), and fluoxetine (5 mM, Sigma) were solubilized in DMSO and diluted for the infection experiments in infection medium. Cell treatments were started 16 h prior to or 2 h post infection.

### MTT assay

MoKi and Vero E6 cells were cultured in the presence of the indicated drug concentrations, the solvent DMSO or with staurosporine (1 µM, Sigma) that served as a positive control for cytotoxic effects. After 24 h of treatment, cell viability was analyzed by adding 3-(4,5-dimethylthiazol-2-yl)-2,5-diphenyltetrazolium bromide (MTT, Sigma) to the cells for 4 h, followed by OD562 measurements. As the reduction of MTT to formazan crystals is strictly dependent to NAD(P)H-dependent oxidoreductase enzymes in metabolically active cells, the colorimetric assay measures the metabolic activity as an indicator of changes in cell viability, cytotoxicity and proliferation.

### Virus and EBOV infection assay

All virus work was carried out in a biosafety level (BSL) 4 environment at the Robert-Koch-Institute. Ebolavirus Zaire strain (origin Makona) was propagated on MoKi cells and was stored at −80°C. The virus stock titre was 2.94×10^7^/mL as determined by standard Tissue Culture Infectious Dose50 (TCID_50_) assay as described previously [24]. For infection experiments, cells grown on 24-well plates (1×10^5^ cells/well), (Greiner Bio-One) were washed with phosphate-buffered saline (PBS, Sigma) and incubated with EBOV diluted in 500 µL of infection medium (cell culture medium supplemented with 2% fetal calf serum) per well at MOI 1 or MOI 10 for 2 h. The virus-containing medium was then removed, cells were washed once with infection medium, and incubation was continued in fresh infection medium for 24 h in total. Infection rates were determined from three randomly acquired images per sample and were expressed as percentages of the cell totals.

### Immunofluorescence staining

Cells were rinsed with PBS^++^ (Sigma) and fixed with 10% paraformaldehyde for 10 min at room temperature (RT), followed by permeabilization with 0.1% Triton X-100 in PBS^++^ (with calcium and magnesium) for 5 min at RT. Cells were blocked with 2% BSA in PBS for 10 min at RT and were subsequently incubated with a mouse monoclonal primary antibody that specifically recognizes the EBOV nucleoprotein (in-house production, 1.08 mg/mL stock concentration) diluted 1:2,000 in 2% BSA/PBS^++^ for 1 h at 37°C or overnight at 4°C. Cells were then rinsed with PBS^++^ and incubated with the goat anti-mouse secondary antibody coupled to Alexa Fluor 488 (115-545-003, Jackson Immuno Research) diluted 1:1000 in 2% BSA/PBS^++^ for 1 h at 37°C. Nuclei were stained with DAPI (Sigma). Cells were rinsed twice with PBS^++^ and stored at 4°C for imaging. Image acquisition of random positions per well was performed using an Evos widefield fluorescence microscope (ThermoFisher) equipped with a 10× air objective. To obtain the cell totals, the DAPI signals were quantified using the Image J software [25] plugin “2D objects counter”. To quantify the infected cells, EBOV nucleoprotein-positive cells were counted manually.

### RNA extraction and qRT-PCR

Cell supernatants were transferred into AVL solution (lysis buffer for purification of viral nucleic acids provided by Qiagen as part of the RNA extraction protocol) and 70% EtOH (vol:vol) was added for virus inactivation. Subsequently, the mix was supplemented with virus-like particles (VLPs) to control for RNA extraction efficiency. Such reference VLPs contain an artificial sequence designed to share no significant homology to any sequence stored in the GenBank repository. Reference VLPs were generated according to standard protocols. Total RNA extraction from the supernatants was performed using the RNeasy Mini Kit (Qiagen) following the produceŕs instructions. For real-time quantification, the AgPath-ID™ One-Step RT–PCR master mix (ThermoFisher) was used on a CFX96 qPCR instrument (BioRad). Primers used were: EBOV VP30 fwd – ACTCCTACTAATCGCCCGTAAG, EBOV VP30 rev – ATCAGCCGTTGGATTTGCT [20], VLP fwd – GGTGATGCCGCATTATTACTAGG, and VLP rev – GGTATTAGCAGTCGCAGGCTT. Detection probes used were: EBOV – FAM-CACCCAAGGACTCGC-MGB and VLP – TexasRed-TTCTTGCTTGAGGATCTGTCGTGGATCG-BHQ1 (MOLBIOL). PCR reactions specific for EBOV and the reference VLPs were run separately to prevent template repression. Ct values of reference VLPs were used as internal reference for RNA extraction efficiency. Ct values for itraconazole-, U18666A-, and fluoxetine-treated samples were compared to the corresponding Ct values of DMSO control samples and viral titres were calculated using a standard curve [26].

### Filipin staining and colocalization analysis

For visualization of endolysosomal compartments, MoKi cells grown on Ibidi slides (8-well chamber) were incubated with 200 nM of Lyso-Tracker Red DND-99 (Molecular Probes, diluted in medium) for 1 h prior fixation with 4% paraformaldehyde in PBS^++^ for 10 min at RT. To visualize cholesterol, fixed cells were incubated with filipin (filipin complex from *Streptomyces filipinensis*; Sigma catalog no. F9765, stock, 2.5 mg/ml in DMSO, diluted 1:2 in 2% BSA/PBS^++^) for 2 h. Confocal microscopy was performed using an LSM 780 microscope (Carl Zeiss, Inc., Jena, Germany) equipped with a Plan-Apochromat 63x /1.4 oil immersion objective. Z-stack series of individual cells were obtained and thresholded prior to Manderś coefficient calculation. To assess the overlap of the lysotracker signals with the filipin signals, Manders’ coefficients were calculated using the ImageJ [25] plugin “JaCoP”. This method indicates the proportion of signal overlap between two channels [27]. Briefly, we calculated the fraction of the total double-positive pixels for each channel and correlated their intensity. The resulting coefficient ranges from 0 (no overlap) to 1 (100% overlap). To generate the cholesterol heatmap, the filipin pixel intensity values of individual images were colour-coded with the gradient of colours ranging from blue (lowest intensity) to white (highest intensity) according to the colour look-up table (LUT).

### Analysis of cellular cholesterol content in Vero and MoKi cells

For quantification of global cellular cholesterol contents, the Amplex Red cholesterol assay kit (Invitrogen) was carried out as described previously [28,29].

### Endosomal pH measurement

To determine endolysosomal pH values, ratiometric fluorescence microscopy was performed as described previously [30]. Briefly, cells were pulsed for 1 h with Oregon green488 (OG488)-labeled 10 kDa-dextran (Thermo Fisher) and Flamma648-labeled 10 kDa-dextran (Biomol), followed by a 1 h chase period. During image acquisition cells were kept in HEPES-buffered Hanks’ balanced salt solution (HBSS, Sigma) at 37°C. Epifluorescence signals were acquired for each of the dyes individually and the mean OG488/ Flamma648 fluorescence ratios were calculated and compared to the calibration curve using standard solutions ranging from pH 4.7 to 6.0.

### TCID_50_ assay

Briefly, cells (3×10^4^cells/ well) were seeded in a 96-well plate, washed twice with PBS^++^ and infected with a 10-fold serial dilution of the virus inoculum for 1 h. After initial infection with the inoculum, the inoculum was removed and cells were cultivated for 7 days in DMEM containing 2% FBS. After incubation, TCID_50_ is calculated by the Spearman & Kärber algorithm as described elsewhere [31]. Additionally, we calculated the focus-forming unit (FFU) per mL by multiplying the TCID_50_ value by the factor 0.69 [24].

### Statistical analysis

The required sample size was estimated via a priori power analysis (G*Power 3.1 [32]). The sample size of each experiment was at least *n* = 3. Data were analyzed with Prism 8.00 (Graph-Pad). Statistically significant differences were evaluated using one-way ANOVA followed by Dunnett’s multiple comparison test or unpaired student’s *t*-test. * p < 0.05, ** *p *<0* *.01, *** *p *<0* *.001, **** *p *≤0* *.0001.

## Results

### Assessing the cytotoxicity of drugs that interfere with NPC1

To exclude cytotoxic effects of the drug treatments, we performed a MTT assay. Similar to what has been reported in human cell lines, itraconazole and U18666A provoked cytotoxicity only at very high concentrations both in MoKi, Vero E6 and A549 cells. In line with our previous observations [17], concentrations of 2 µg/mL used in the infection experiments had no detectable effects on cell viability (Suppl. Figure 1A).

### Treatment with itraconazole and U18666A results in cellular cholesterol imbalance

**Figure 1.**
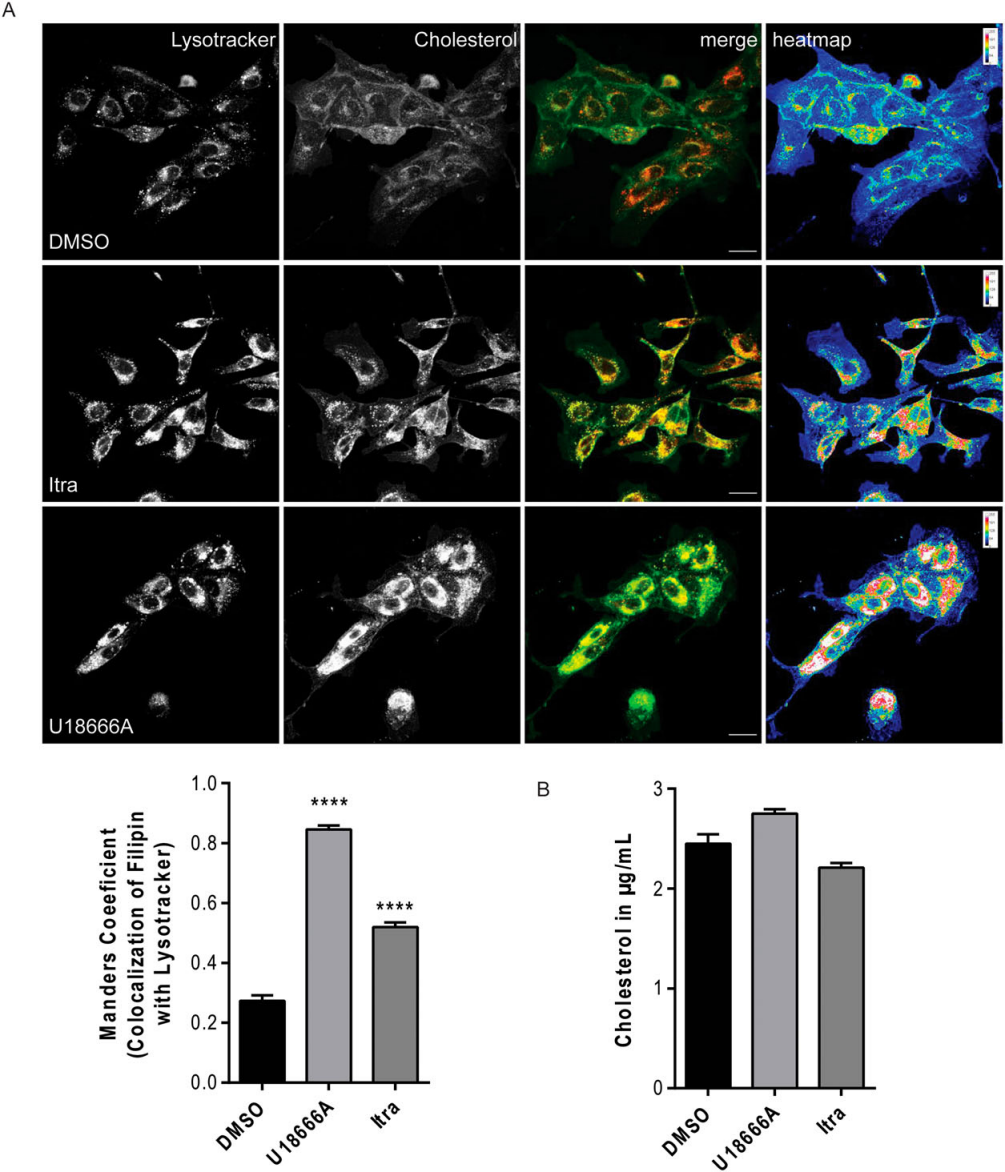
Treatment with U18666A or itraconazole increases endolysosomal cholesterol storage in bat-derived cells. (A) Cellular cholesterol pools in MoKi control cells (DMSO-treated) or MoKi cells treated for 16 h with itraconazole (Itra, 2 µg/mL) or the NPC1 inhibitor U18666A (2 µg/mL). Endolysosomes were stained with the organelle-specific marker lysotracker and unesterified cholesterol was visualized using filipin. Heatmaps were generated by colour-encoding filipin-positive pixels according to their intensity values. Representative 2D maximum intensity projections of entire z-stacks obtained by confocal imaging of 3 individual experiments are shown. Scale bar, 20 µm. (B) Overlaps of filipin/LysoTracker signals within the stacks were analyzed by calculating Manders’ coefficients. Bar graphs represent means ± SEM of 9 stacks, with 0 indicating no overlap, and 1 indicating perfect overlap. (C) Global cellular cholesterol levels. Data are expressed as mean cholesterol concentrations (µg/mL) ± SEM from five independent experiments. Statistically significant differences were assessed by one-way ANOVA followed by Dunnett’s multiple comparison test. *p*-values ≤ 0.05 were considered statistically significant. ****p ≤ 0.0001.

Our previous work already confirmed that itraconazole, similar to the direct NPC1 inhibitor U18666A, inhibits endolysosomal cholesterol export in the monkey-kidney Vero-E6 cells [16,17]. To elucidate whether both drugs can also influence cellular cholesterol distribution in bat-derived cells, we compared cellular cholesterol pools in MoKi cells treated for 16 h with itraconazole or U18666A with the solvent-treated control cells via confocal microscopy. The endolysosomal compartments were visualized with the organelle-specific dye LysoTracker and free cellular cholesterol was stained with filipin, a cholesterol-binding fluorescent macrolide [33]. The digital images were pseudocolored according to the filipin signal strengths, resulting in heatmaps of cholesterol distribution within the cells. Notably, we observed a strong cholesterol accumulation in the endolysosomal compartment when cells were treated with itraconazole and U18666A (Figure 1A). The elevated amount of endolysosomal cholesterol was confirmed quantitatively by calculating the Manders’ colocalization coefficient (MCC [27]), a method to determine the degree of signal overlap (Figure 1A). To assess whether these drugs also affect global cellular cholesterol levels, we additionally quantified total cellular cholesterol contents using Amplex Red, a widely used fluorometric assay to quantify cholesterol [34]. As seen previously in human cells [17], cellular cholesterol contents were not significantly altered in itraconazole or U18666A-treated MoKi cells compared to control cells (Figure 1B), indicating that both drugs trap cellular cholesterol contents in endolysosomes without changing the total cellular cholesterol levels in MoKi cells.

Because EBOV fusion with the endosomal membrane occurs in a pH-dependent manner [10,35], we assessed whether drug treatment altered the endosomal pH value. Similar to what we observed in human cells [16,17], quantitative ratiometric fluorescence microscopy [16] revealed significant changes in endosomal pH values in U18666A-treated MoKi cells, while itraconazole treatment had no detectable impact on endosomal pH values (Suppl. Figure 2).

### Targeting Niemann-Pick C1 (NPC1) with itraconazole or U18666A exerts antiviral potential against EBOV infection

**Figure 2.**
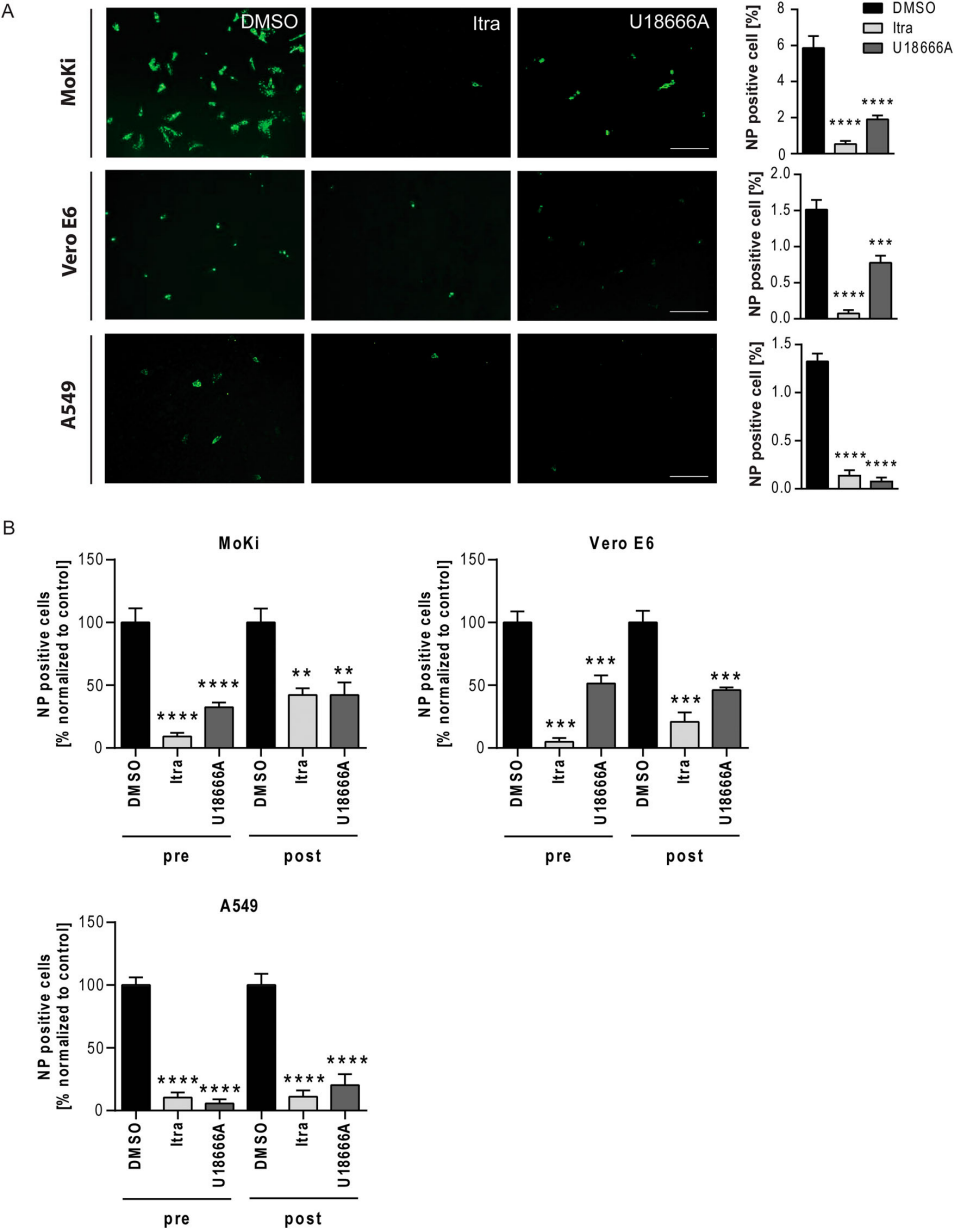
Targeting NPC1 with the clinically licensed drug itraconazole or U18666A impairs EBOV infection. (A) Representative images of infected and NP-specific immunostained cells. Cells were infected using EBOV (Zaire, MOI 1) for 24 h and treated with itraconazole (Itra, 2 µg/mL) or U18666A (2 µg/mL) 16 h prior to infection. DMSO served as control. Immunostaining was performed using an anti-nucleoprotein (NP, monoclonal mouse) primary antibody in combination with a goat anti-mouse Alexa 488 coupled secondary antibody (green). Image acquisition was carried out using the GFP channel of a wide field fluorescent microscope equipped with a 10× objective. Scale bar, 400 µm. NP positive cells were expressed as percentage of total cell counts. (B) Percentage of NP-positive cells in the respective samples normalized to control (set to 100). Cells were either pre-treated (pre) for 16 h, or treated 2 h post infection (post) with the respective drugs. Bars show the means ± SEM of nine independent experiments. Data were analyzed for statistically significant differences with one-way ANOVA followed by Dunnett’s multiple comparison test; *p*-values ≤ 0.05 were considered statistically significant. **p ≤ 0.01, ***p ≤ 0.001, ****p ≤ 0.0001.

Next, we assessed the antiviral capacity of itraconazole and U18666A treatment against the EBOV strain Zaire. To simulate both prophylaxis and therapeutic treatment, MoKi and Vero E6 cells were treated with itraconazole or U18666A either 16 h before infection or 2 h post infection with the EBOV strain Zaire (1 MOI, 24 h infection period). DMSO-treated cells served as controls. Infection rates were determined via microscopy-based analysis. DAPI staining was used to mark cell nuclei, EBOV-infected cells were identified by staining the viral nucleoprotein (NP) (Figure 2A). We observed a different susceptibility of the cell lines towards EBOV infection (MoKi: ∼ 6%, Vero E6: ∼ 1.5%, and A549: ∼ 1.5% infection rate, Figure 2A). To allow easier comparison between the antiviral capacity of itraconazole and U18666A treatment in the different cell lines, we presented the subset of infected cells expressed as percentage of infected cells normalized to infected control cells (Figure 2B). As shown in Figure 2A and B, itraconazole treatment reduced viral infection levels up to 90% in all three tested cell lines, while U18666A treatment was only able to reduce viral infection to a similar extent in the human cell line A549 when administered 16 h prior infection (Figure 2B). Additionally, we assessed the antiviral effect of both drugs in the post-infection scenario (2 h post infection). In the human cell line A549, both drugs caused a significant reduction in viral infection up to 90% compared to the control condition, while the antiviral effect was less pronounced in MoKi and Vero E6 cells (Figure 2B). Together, these findings underscore the suitability of NPC1 as a druggable target to block EBOV cell entry and identify the clinically licensed antifungal itraconazole as a promising candidate for repurposing.

### Pharmacological intervention with the acid sphingomyelinase inhibitor fluoxetine impairs EBOV infection

**Figure 3.**
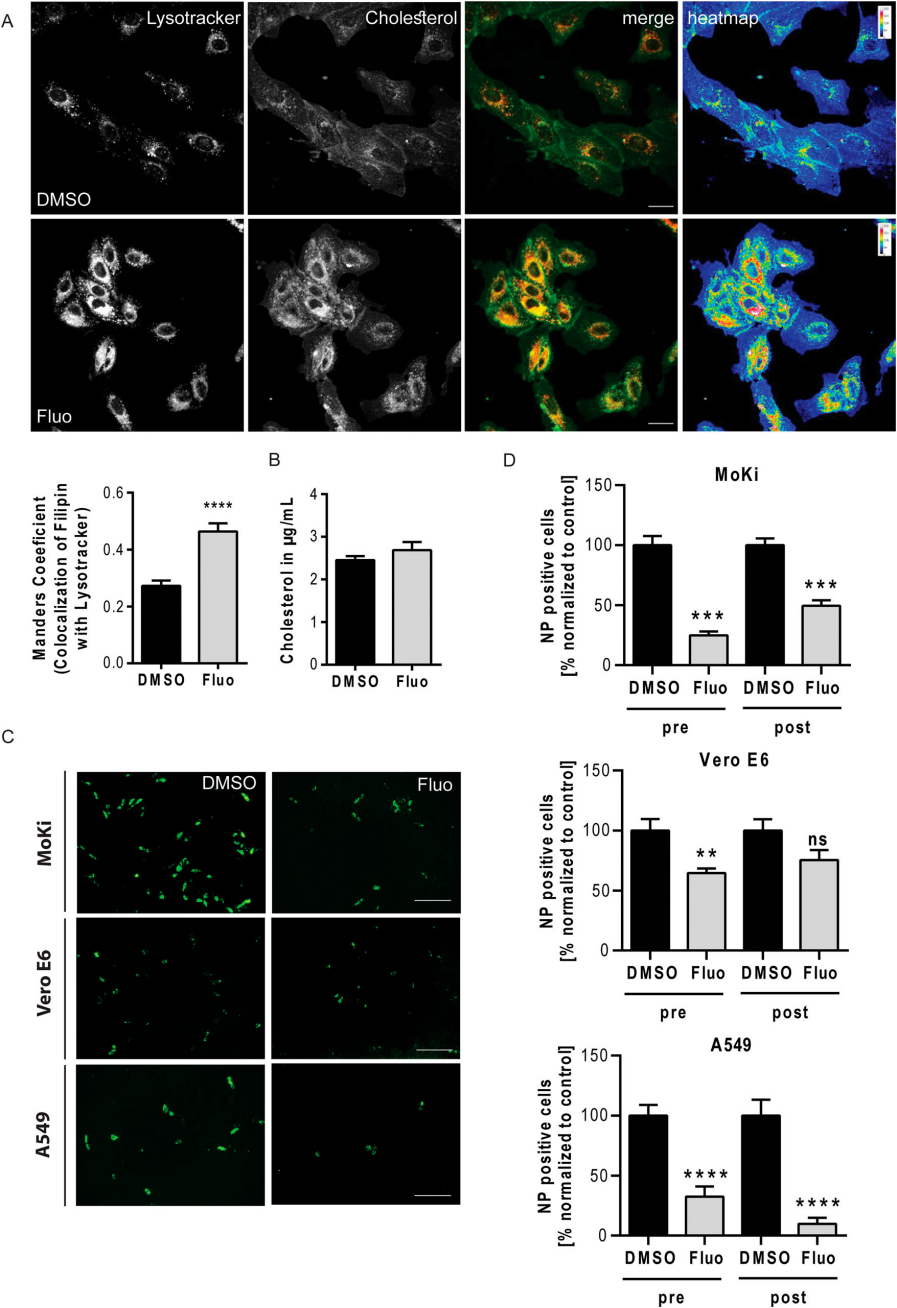
Interfering with the acid sphingomyelinase activity via fluoxetine impairs EBOV infection. (A) MoKi cells were treated for 16 h with either the solvent DMSO or fluoxetine (5 µM). Lysotracker was used to visualize the endolysosomal compartment and cellular cholesterol was stained with filipin. Representative 2D maximum intensity projections of entire z-stacks and heat maps with the filipin-positive pixels colour-encoded according to their intensity values are presented. Scale bars, 20 µm. Manders’ colocalization coefficients of LysoTracker signals overlapping with filipin were quantitated from z-stacks. Bar graphs represent means ± SEM of three independent experiments. (B) Global cellular cholesterol levels. Data are expressed as mean cholesterol concentrations (µg/mL) ± SEM from five independent experiments. (C) Representative images of NP-positive cells. MoKi, Vero E6, and A549 cells were infected using EBOV (Zaire, MOI 1) for 24 h and treated 16 h prior to infection (pre) with fluoxetine (5 µM), respectively. DMSO served as control. Infected cells were detected via immunostaining using an anti-nucleoprotein (NP) antibody. Infection rates were calculated as percentages of NP-positive cells from total cell amount. Scale bar, 400 µm. (D) Quantitative analysis of infection rates. Cells were either pre-treated (pre) for 16 h, or treated 2 h post infection (post) with fluoxetine. Bar graphs represent means ± SEM of nine independent experiments, with the samples normalized to control (set to 100%). Data were analyzed with unpaired t-test; **p ≤ 0.01, ***p ≤ 0.001, ****p ≤ 0.0001.

Our recent findings revealed that functional inhibitors of the endosome-residing acid sphingomyelinase (FIASMA) including the antidepressant fluoxetine [23] also interfere with endolysosomal cholesterol balance without directly targeting NPC1, an effect that could be exploited to impair IAV and SARS-CoV-2 infections in the cell culture model [16]. As shown in Figure 3AB, Suppl. Figure 1B, elevated endosomal cholesterol levels were also seen in MoKi cells treated with fluoxetine, pointing at a similar mechanism of cellular cholesterol handling operating in the two species. We next assessed the antiviral potential of fluoxetine treatment against EBOV infection in all three cell lines. While the antiviral effect of fluoxetine treatment was less pronounced in the two zoonotic cell culture models (Vero E6: up to 30%, and MoKi: up to 75%), levels of infected cells were strongly impaired in the fluoxetine-treated human lung carcinoma cell line A549 (reduction of NP-positive cells up to 90%) (Figure 3CD). Thus, the clinically well-established FIASMA fluoxetine showed a promising antiviral potential against EBOV infection.

### EBOV propagation is altered upon treatment with U18666A, itraconazole or fluoxetine

**Figure 4.**
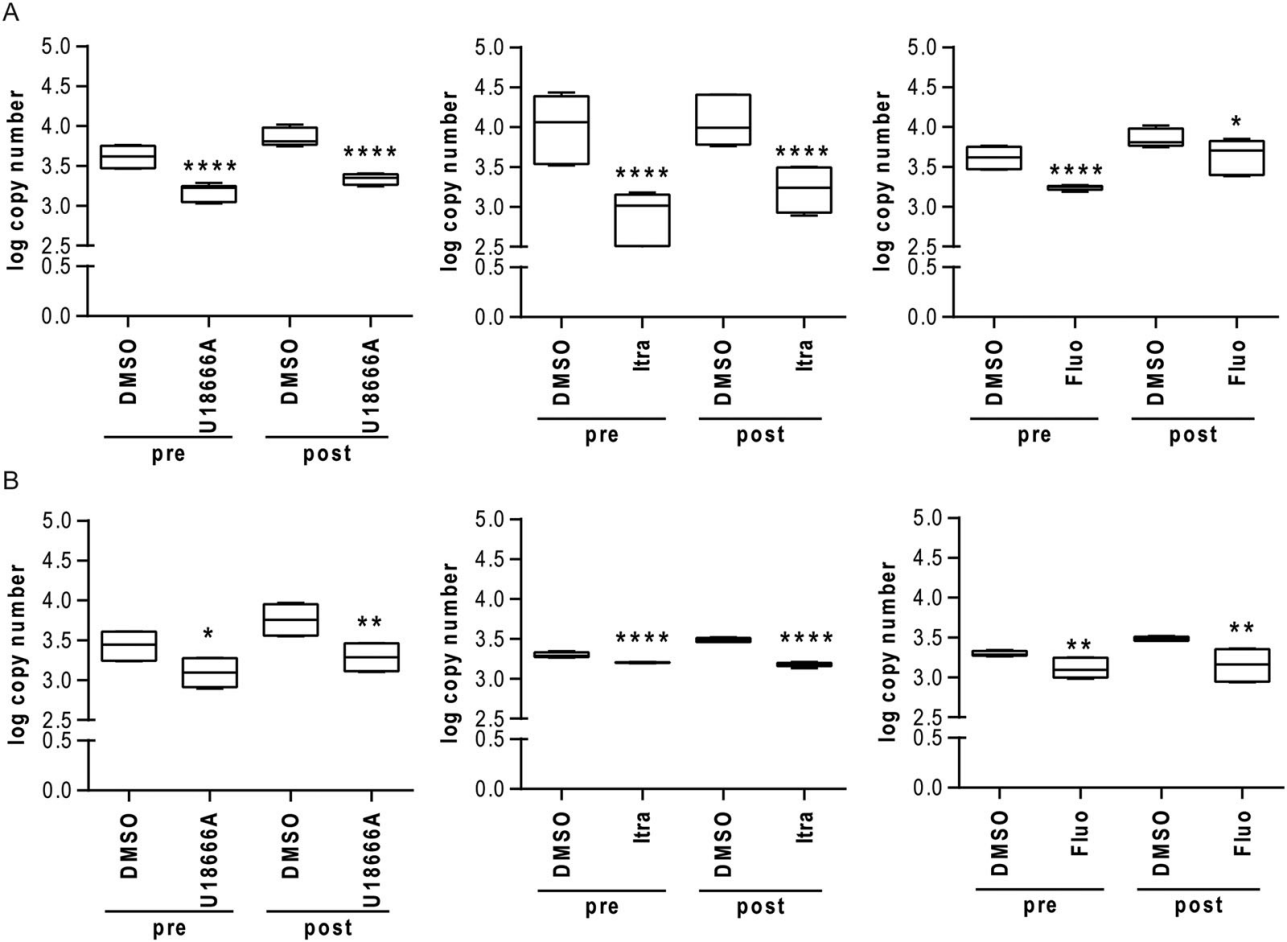
Treatment with U18666A, fluoxetine or itraconazole reduces viral particle release in MoKi and A549 cells. (A) MoKi and (B) A549 cells were treated for 16 h prior to infection (pre) or 2 h post infection (post) with either DMSO, U18666A (2 µg/mL), itraconazole (2 µg/mL) or fluoxetine (5 µM). Copy numbers of viral particles were determined in supernatants by RT-PCR. Box-and-whiskers plots (minimum to maximum) indicative of six samples of three independent experiments. Data were analyzed with One-way ANOVA; *p ≤ 0.05, **p ≤ 0.01, ****p ≤ 0.0001.

We next assessed the effect of impaired cholesterol on the propagation of newly formed virus particles. Thus, we collected the supernatant from EBOV-infected MoKi and A549 cells that were treated with U18666A, itraconazole or fluoxetine either 16 hrs prior to or 2 h post infection. The efficiency of viral RNA extraction was controlled by addition of reference VLPs encoding for an artificial sequence (to be published elsewhere). RT–PCR was performed individually for the VP30 gene of EBOV and the reference sequence and their copy numbers were determined using a standard curve (Suppl. Figure 3) (Figure 4). Treatment with either itraconazole, U18666A, or fluoxetine resulted in significant reduced viral genome copy numbers in the supernatant of MoKi and A549 cells (Figure 4). Together with the NP staining (Figures 2 and 3), the reduction in viral genome copy numbers in the zoonotic MoKi and the human A549 cell line showed the beneficial antiviral effect of drugs targeting the cholesterol homeostasis.

### Assessing the antiviral potential at higher initial infectious dose

**Figure 5.**
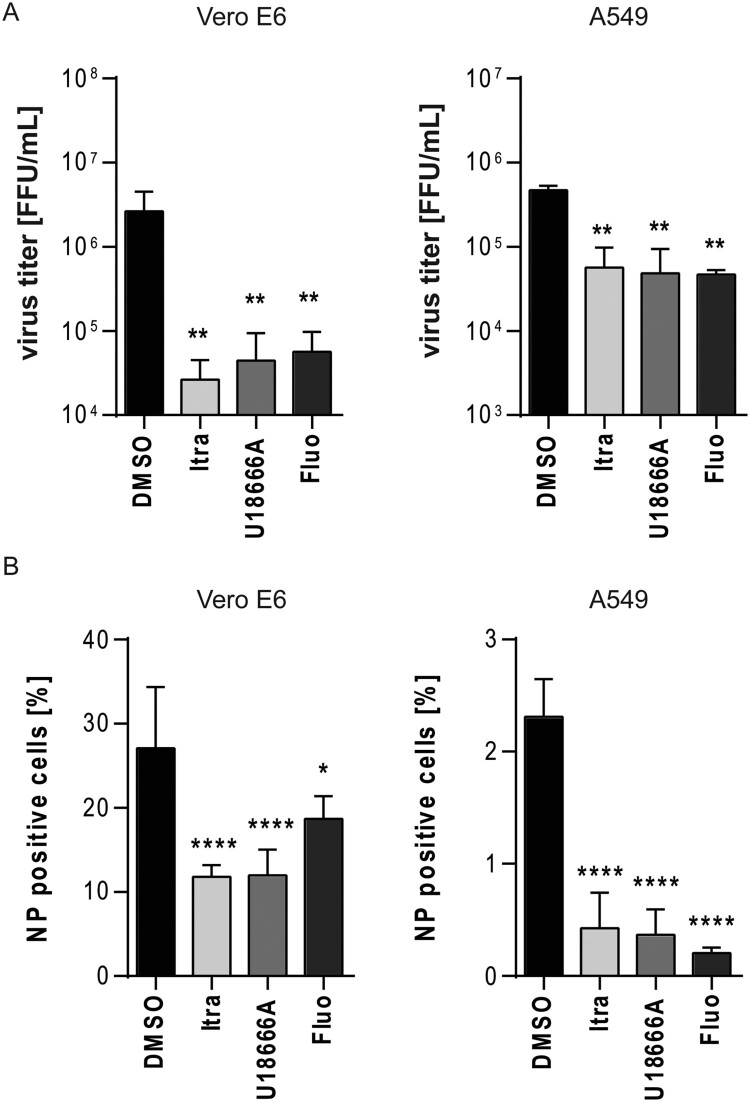
Evaluation of the antiviral potential at higher initial infectious dose. Vero E6 and A549 cells were infected with EBOV (Zaire, MOI 10) for 24 h and treated with itraconazole (Itra, 2 µg/mL), U18666A (2 µg/mL), or fluoxetine (Fluo, 5 µM) for the entire infection period. DMSO served as solvent control. (A) Viral titres were determined by TCID_50_ assay and FFU were calculated. (B) Quantification of viral infection levels via immunofluorescence assay. Numbers of NP positive cells were expressed as percentages of total cell counts. Bars show the means ± SEM of five independent experiments. Data were analyzed for statistically significant differences with one-way ANOVA followed by Dunnett’s multiple comparison test; *p*-values ≤ 0.05 were considered statistically significant. *p ≤ 0.05, **p ≤ 0.01, ****p ≤ 0.0001.

Because the initial infectious dose is known to be a decisive factor in determining the fatal outcome of EBOV infections, we next evaluated the antiviral properties of the three compounds at a higher initial viral dose. Vero E6 and A549 cells were infected at MOI of 10, and were treated with the respective compounds for 24 h. Viral titres were subsequently determined by TCID_50_ and FFU were calculated as described. While the solvent treated cells yielded viral titres up to 3×10^6^ FFU/mL in Vero E6 and 7×10^5^ FFU/mL in A549 cells, significantly viral titre reductions of at least 1 log step in A549 or ∼ 1.5 log steps in Vero E6 cells were observed with all three treatments (Figure 5A). Additional evaluation of infection levels via microscopy confirmed the antiviral effects of the compounds at higher MOI (Figure 5B). Interestingly, Vero E6 cells were more susceptible than A549 cells (Vero E6: ∼28%; A549: ∼2%, Figure 5B), which is in line with the higher infectious particle released in Vero E6 cells. These data showed that the antiviral effect of the compounds is not restricted to a low initial infection dose.

### Dose-dependent anti-EBOV activities of itraconazole, U18666A, and fluoxetine

**Figure 6.**
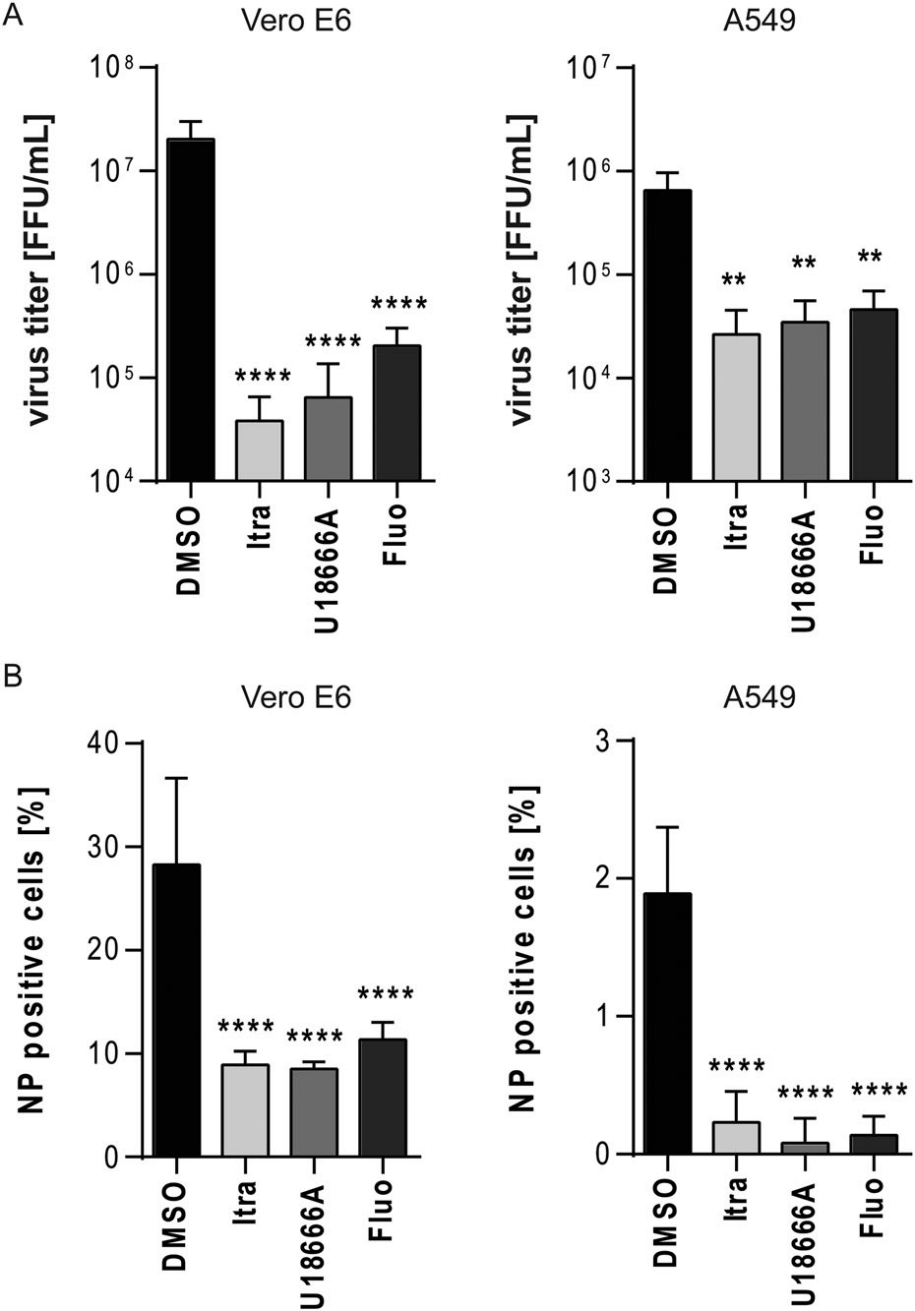
Dose-dependent anti-EBOV activities of drugs acting on the cellular cholesterol homeostasis. Vero E6 and A549 cells were infected using EBOV (Zaire, MOI 10) for 24 h and treated with itraconazole (Itra, 10 µg/mL) or U18666A (10 µg/mL) or fluoxetine (Fluo, 20 µM) for the entire infection period. DMSO served as solvent control. (A) Viral titres were determined by TCID_50_ assay and FFU calculated. (B) Quantification of viral infection levels via immunofluorescence assay. NP positive cells were expressed as percentage of total cell counts. Bars show the means ± SEM of five independent experiments. Data were analyzed for statistically significant differences with one-way ANOVA followed by Dunnett’s multiple comparison test; *p*-values ≤ 0.05 were considered statistically significant. **p ≤ 0.01, ****p ≤ 0.0001.

To evaluate whether higher concentrations of the compounds strengthen the anti-EBOV effects in Vero E6 and A549 cells, we increased the concentrations of the respective compounds (10 mg/mL itraconazole, 10 mg/mL U18666A; 20 µM fluoxetine), subsequently infected cells with a high initial viral dose (MOI of 10), determined viral titres, and quantified the infection levels via microscopy. Indeed, for each drug and both cell lines, the higher doses caused a significantly stronger reduction in viral titres compared to the lower dose treatments (Figures 5A and 6A). The higher dose of itraconazole and U18666A decreased viral titres up to 2.5 log steps, while the elevated fluoxetine concentration reduced viral titres up to 2 log steps (Figure 6A). Treatment with 20 µM of fluoxetine was able to significantly reduce the amount of NP positive cells while this was not observed in cells treated with increased U18666A or itraconazole concentrations (Figures 5B and 6B). However, all three treatments led to a notable reduction in NP positive cells when compared to the control (Figure 6B). Thus, for all three compounds that act on the endolysosomal cholesterol homeostasis increased concentration can improve the antiviral capacity and might help to circumvent EBOV infection in a safe antiviral window (Suppl. Figure 1).

## Discussion

A characteristic of emerging viruses like EBOV is their ability to adapt to the host, switch to a new host, and to evolve strategies to escape antiviral measures. Thus, epidemic outbreaks caused by EBOV appeared with increasing numbers of morbidity and fatality rates [1]. Since antiviral strategies to combat EBOV infections are very limited and strain-dependent, we investigated whether the repurposing of the clinically licensed therapeutics itraconazole and fluoxetine have potential in EDV precaution and antiviral treatment. Both drugs were recently found to be antiviral against influenza virus and SARS-CoV-2 infection [16,17,36].

While itraconazole, similar to the NPC1 inhibitor U18666A, targets the endosomal cholesterol transporter Niemann-Pick C1 (NPC1), the antidepressant fluoxetine acts on the lysosome-residing acid sphingomyelinase [14,23]. Pharmacological inhibition of cellular proteins like NPC1, the main entry receptor of EBOV, interferes with an early stage of EBOV life cycle [9,10,12]. Treatment with the NPC1 inhibitors itraconazole and U18666A or with the FIASMA fluoxetine resulted in endolysosomal cholesterol sequestration in cells of two different zoonotic species (MoKi and Vero E6 cells), and in the human A549 cell line that may either direct or indirect impair viral fusion and/or uncoating at the endolysosomal membrane. The infection rates determined through immunostaining of EBOV NP were significantly reduced in almost all applied treatments upon pre-incubation for 16 h or post-infection treatment (Figures 2 and 3). Of note, both NPC1-targeting drugs (itraconazole and U18666A) resulted in noticeable reduction of NP-positives cells, underlining that NPC1 is a druggable target in EBOV infection scenario (Figure 2). Although reduction in numbers of EBOV-infected zoonotic cells were less pronounced, treatment with the acid sphingomyelinase inhibitor fluoxetine was able to impair EBOV infection to similar levels in the human lung cell line A549 (Figure 3). These findings are in accordance with previous studies demonstrating the antiviral activity of itraconazole and fluoxetine against IAV and SARS-CoV-2 infection *in vitro* (Calu-3, Vero E6, A549, and A431) and *in vivo* (mice) [16,17]. The effect of cholesterol imbalance is not only restricted to an altered viral entry but rather results in a reduction of replication and particle production which is reflected by the decreased abundance of EBOV genomes and thus viral particles in the supernatant of infected zoonotic MoKi (derived from the supposed zoonotic reservoir species for EBOV, *Mops condylurus* [20,37]) and human A549 lung cells (Figure 4). Although MoKi cells harbour a moderate expression level of the suggested entry receptor NPC1 compared to HEK293 or HeLa cells [20], treatment with the NPC1 inhibitor U18666A was very effective in counteracting EBOV infections, arguing for NPC1 as the main entry receptor in the different mammalian species [20]. These data are supported by the observation that NPC1 knockout mice remained completely resistant to EBOV infection [38], supporting its possible use as a therapeutic target. The antiviral mechanism of U18666A and itraconazole might not be only restricted to the direct inhibition of NPC1 but rather as a result of cholesterol sequestration within the endolysosomal system impeding viral fusion, which is supported by our observation that either the NPC1-targeting drugs itraconazole and U18666A or the FIASMA fluoxetine can abrogate viral entry by causing a massive cholesterol accumulation. Inhibition of the endolysosome-resident acid sphingomyelinase (ASM) via fluoxetine leads to an abolished conversion of sphingomyelin into ceramide [23]. By blocking the generation of ceramide via ASM, fluoxetine dramatically alters the biophysical membrane properties as evidenced by the fluoxetine-induced endolysosomal cholesterol build-up which interferes with EBOV entry (as shown in Figures 3, 5 and 6). A second contributing antiviral effect of fluoxetine and the direct NPC-1 inhibitors U18666A and itraconazole is caused by a change in lipid composition not only of cellular membranes including the plasma membrane but also of the host-derived viral envelope which is known to affect EBOV infectivity [39].

The complex entry pathways utilized by EBOV not only depend on the main EBOV receptor NPC1 [9,10,12] but rather employ multiple factors involved in endosome and lysosome biogenesis and maturation [9]. Like most enveloped viruses [40], EBOV accessorily exploits the drop in pH encountered in the endolysosomal compartment as a trigger for viral envelope-endosomal membrane fusions which is needed for the cytosolic release of the viral genome [41]. Indeed, blocking of endolysosomal acidification by bafilomycin A1-mediated inhibition of the vacuolar-type H^+^ -ATPase (V-ATPase) prevents EBOV entry [41]. Because low endolysosomal pH is crucial for the fusion of viral and endosomal membrane, we additionally analyzed the influence of the treatments (itraconazole, U18666A and fluoxetine) on endolysosomal pH in zoonotic MoKi cells. Of note, endolsosomal pH values were altered in MoKi cells upon treatment with U18666A and fluoxetine (Suppl. Figure 2), arguing that both drugs also interfere with the acidification of the endolysosomal compartment. It is likely that the acidic environment is necessary to activate the endosomal cathepsins which proteolyze the viral glycoprotein (GP) [42–44]. However, the precise mechanism whether cathepsin cleavage of GP itself causes some conformational changes that trigger the fusogenic conformation [43] or if the cathepsin-driven cleavage activates GP for triggering by an additional unknown cellular factor is not fully understood. Nevertheless, drug-induced changes in the environment of cellular compartments involved in the entry process of EBOV is a suitable antiviral strategy. A conclusion that is supported by an earlier study showing that targeting macropinocytosis with EIPA [5-(N-ethyl-N-isopropyl) amiloride], an inhibitor of the Na^+^/H^+^ exchanger that specifically inhibits macropinocytosis [45,46], can also abrogate EBOV infection [47]. Interfering with the complex regulatory circuits of endolysosomal lipid balance and functionality might serve as a feasible target for anti-EBOV therapy and other pathogens (like influenza viruses, [29]) with functionally similar entry pathways.

We further observed a higher antiviral efficacy of itraconazole when compared to U18666- or fluoxetine-treated cells (Figures 2, 5, and 6). While U18666A induced significantly higher endolysosomal cholesterol accumulation than itraconazole, the latter might also act through modulating signalling pathways that are required for successful virus replication and propagation. A comparison of host gene expression signatures upon infection with the EBOV strain Makona revealed the importance of mammalian target of rapamycin (mTOR) signalling in EBOV infection [48,49]. A screening of a kinase inhibitor library showed that several kinases are activated through EBOV life cycle and pharmacological intervention via inhibitors suppressed viral replication [50]. Of note, treatment with the epidermal growth factor receptor (EGFR) inhibitor Gefitinib altered the biology and subvesicular localization of NPC1, rendering these compartments non-conducive to EBOV entry [50]. Itraconazole as a known inhibitor of mTOR signalling [51] and modulator of Vascular Endothelial Growth Factor (VEGF), belonging to the receptor tyrosine kinase family, or of the Wnt/β-catenin signalling pathways [52,53] might exerts additional antiviral effects through modulation of these pathways.

The antiviral effect of all three tested pharmaceuticals was even robust at a higher initial infectious dose, clearly showing that they also interfere with viral infection at higher MOIs. Additionally, we observed a significant dose-dependency of the three pharmaceuticals in reducing viral titres, while the levels of infected cells are only slightly altered (Figures 5 and 6). These data are in line with the observation of a dose-dependency of the compounds in the capacity to reduce influenza and SARS-CoV-2 titre [16,17,36].

Because these host-oriented drugs are acting on the general entry pathways utilized by viruses, they are less prone to induce antiviral resistances and immune escape compared to direct antiviral drugs such as the newly developed monoclonal antibody REGN-EB3 (Inmazeb [54]) which could lead to immune escape variants in future. A bottleneck in translating therapeutic strategies from in vitro to clinical approaches is the bioavailability of drugs that need to reach plasma level concentrations which exert the antiviral effects. While the bioavailability after oral application of itraconazole is low because of the low water solubility of this highly lipophilic compound and its weak absorption from the gastrointestinal tract [55,56], a recommended daily dose of 200–400 mg·day^−1^ (used in prophylaxis and treatment of fungal infections) or 600 mg·day^−1^ in case of severe infections reaches plasma concentration of > 500 μg·L^−1^ (when administered 200 mg·day^−1^) well above the concentration that were required for anti-Ebola virus activity in this study [57,58]. In contrast to itraconazole, the bioavailability of orally administered fluoxetine is high, leading to plasma levels of 350 μg·L^−1^ after 2 weeks and up to 1055 μg·L^−1^ when administered for longer treatment regime (20 mg·day^−1^ fluoxetine [59,60]). These reported plasma levels are sufficient enough to establish the antiviral effect against EBOV.

Although pharmacokinetic and safety profiles for itraconazole and fluoxetine are available (information available at Drugs.com 2021), their clinical use and appropriate treatment strategy should rely on patient's genetic disposition, physiological or pathophysiological conditions. The careful administration of drugs should exclusively rely on the medical advice. However, the large variety of azole such as itraconazole or posaconazole and FIASMA pharmaceuticals offer a toolbox of potential antivirals for host-directed therapy that can be administered depending on clinical implications and counteract EBOV and ameliorate EDV severity.

## Supplementary Material

Supplemental Material

Supplemental Material

Supplemental Material
